# The relationship between athletes’ self-esteem and perceived social support: the mediating effect of decentering and the moderating effect of training years

**DOI:** 10.3389/fpsyg.2025.1617429

**Published:** 2025-10-29

**Authors:** Xianbang Huang, Ling Li

**Affiliations:** ^1^Faculty of Sports Training, Xi'an Physical Education University, Xi'an, China; ^2^Faculty of Sports and Health Sciences, Xi'an Physical Education University, Xi'an, China

**Keywords:** athletes, self-esteem, decentering, perceived social support, training years

## Abstract

**Objective:**

This study aimed to investigate the relationship between athletes’ Self-esteem(SE) and The Perceived Available Support in Sports Questionnaire(PASS-Q), with a particular focus on the mediating role of Decentering and the moderating effect of training years.

**Methods:**

A total of 354 athletes (198 males and 156 females) from various sports were surveyed using the Rosenberg Self-Esteem Scale, The Decentering Scale for Sport, and The Perceived Available Support in Sports Questionnaire. Correlation analysis, regression analysis, and PROCESS macro modeling were employed to examine the hypothesized relationships.

**Results:**

(1) Athletes’ SE is significantly positively correlated with Decentering and PASS; (2) Decentering played a mediating role in the relationship between SE and PASS; (3) Training years moderated the direct effect of SE on PASS, such that the effect was stronger among athletes with longer training years.

**Conclusion:**

SE not only directly promotes athletes’ PASS but also indirectly enhances it through Decentering. Furthermore, training years serve as a boundary condition for this relationship. These findings provide theoretical insights and practical implications for improving athletes’ psychological resilience and social adaptation.

## Introduction

In competitive sports, athletes’ success in achieving outstanding performance and results depends not only on their physical abilities but also on their psychological well-being (Reardon et al., 2019). Evidence suggests that support from family, friends, or coaches helps athletes withstand competitive pressure, maintain stable performance (Kristiansen and Roberts, 2010), and reduce aggressive tendencies (Jaureguizar et al., 2024). Such support serves as a robust protective factor for athletes’ mental health, substantially lowering the risk of psychological problems, and is of inestimable value for sustaining athletes’ psychological stability and enhancing performance. Social support consists of two essential components: perceived social support and received social support. Prior research has argued that the “perception” of support is more critical than its actual receipt, meaning that perceived social support may exert a more positive influence on individuals than received support (Lakey and Orehek, 2011). Perceived social support refers to an individual’s subjective judgment regarding the availability of support when needed(Lakey, 2010; Uchino, 2009). Empirical findings indicate that higher levels of PASS contribute to psychological well-being (Cho et al., 2020; DeFreese and Smith, 2014; Poudel et al., 2020). For injured athletes in particular, perceived support has a salient effect on their coping, persistence in rehabilitation, and emotional recovery (Clement and Shannon, 2011), further motivating them to pursue enhanced performance (Brown et al., 2018; Forsdyke et al., 2022). At a fundamental level, PASS has been identified as a core psychological resource: it not only buffers the impact of stress but also strengthens resilience and subjective well-being, making it an indispensable element in the protective mechanisms of athletes’ mental health (Liu et al., 2023).

### Self-esteem and perceived available social support

Self-esteem (SE) refers to an individual’s overall cognitive and emotional evaluation of their self-worth, reflecting a sustained and stable level of self-acceptance. It was formally defined by Rosenberg (Rosenberg, 1965). As a core psychological resource, SE influences not only self-cognition and emotional regulation but also plays a pivotal role in the establishment and maintenance of social relationships. For athletes, the level of SE may directly affect their sensitivity to, and integration of, external signals of support (Li et al., 2023). According to sociometer theory (Leary and Baumeister, 2000), individuals with high SE may exhibit lower sensitivity to social value alerts, and a reduction in defensive social monitoring makes it easier for them to perceive support from various sources. Conversely, individuals with low SE tend to have a relatively lower capacity to perceive support. Research by Ikiz and Cakar has confirmed the validity of this theory (Ikiz and Cakar, 2010). Additionally, studies have shown a close relationship between SE and PASS (Kurniawan et al., 2023), and similar findings have been observed in research conducted in China (Liu et al., 2021).

### The mediating role of decentering

Decentering refers to an individual’s ability to regard thoughts, emotions, and bodily sensations as transient mental events rather than linking them to their actual athletic performance or ability(Zhang et al., 2016). It reflects the capacity to disengage from and let go of negative emotions and thoughts, thereby enabling athletes to focus their attention on the task at hand rather than on their own emotions (Zhang et al., 2016). Structurally, decentering represents a specific mode of cognitive processing (Fresco et al., 2007); functionally, it emphasizes “maintaining distance from one’s thoughts” (Bernstein et al., 2015). Research has shown a close relationship between decentering and mindfulness. Mindfulness is typically defined as an intentional, present-moment, non-judgmental form of attention, emphasizing an individual’s open acceptance of current experiences (Kabat-Zinn, 1990). Zhang et al.in developing the Decentering, highlighted that decentering and mindfulness are distinct yet related constructs: while mindfulness emphasizes sustained attentional regulation, awareness, and acceptance, decentering focuses on the decentered perspective and cognitive distancing from internal experiences emphasized that decentering and mindfulness are independent yet related psychological concepts. Mindfulness focuses more on sustained attention regulation, awareness, and acceptance, while decentering emphasizes the decentring of internal experiences and cognitive distancing (Zhang et al., 2016). In other words, mindfulness, by observing without judgment, helps individuals detach from over-identifying with their thoughts, thereby reducing self-centeredness (Hayes-Skelton and Lee, 2020).

Previous research has shown that individuals’ levels of SE are closely associated with their levels of mindfulness (Chandna et al., 2022; Rasmussen and Pidgeon, 2011; Stenhaug and Solem, 2024). Although no study to date has directly examined the relationship between SE and decentering in athletic samples, it can be inferred that such a link may exist, given that mindfulness training has been shown to foster decentering. Existing research has demonstrated a strong correlation between positive emotions and athletes’ performance (Weinberg and Gould, 2023), and the ability to regulate negative emotions is critical (Goñi et al., 2024). Based on the broaden-and-build theory, such positive emotions have the capacity to expand an individual’s cognitive and attentional scope, gradually building enduring psychological and social resources. High self-esteem is often associated with increased positive emotional experiences, such as enhanced emotional regulation ability (Brown and Marshall, 2001), or a greater tendency to express and experience positive emotions, or a greater tendency to express and experience positive emotions (Caprara et al., 2022). These positive emotions prevent individuals from excessively focusing on self-evaluation or threatening information and instead enable the development of the ability to decenter from emotions and thoughts. This ability helps individuals maintain psychological flexibility when facing negative emotions or stressful thoughts, thereby becoming more open to receiving and recognizing external supportive information (Fredrickson, 2001). Therefore, it can be inferred that SE, through the broadened effects of positive emotions, enhances the perception and construction of social support via the mechanism of decentering. Based on this, we hypothesize that decentering mediates the relationship between athlete SE and perceived social support.

Competitive sports are inherently characterized by high pressure and high risk, and athletes inevitably experience errors, injuries, and performance fluctuations. When athletes become excessively entangled in self-blame or negative evaluations, they are prone to heightened anxiety, distraction, and even reduced motivation (Kang and Gong, 2024; Minichiello et al., 2024). Decentering serves as an important psychological resource in this context: by viewing negative thoughts arising in training and competition from a distanced perspective, athletes can avoid the rigid cognitive pattern of equating performance with self-worth, thereby reducing perseverative responses to adverse events and maintaining psychological stability and competitive focus. Within the Chinese cultural context, the function of decentering is particularly salient. Chinese athletes often grow up in a collectivist environment, where self-worth is heavily influenced by external evaluations, collective honor, and coaches’ expectations. In such a cultural setting, athletes are more likely to internalize external feedback as self-evaluation, and poor performance may consequently trigger excessive self-blame and psychological distress (Wang and Lian, 2025). The ability to decenter may help athletes detach from a singular external evaluation system, adopt a more objective and accepting understanding of the self, and enhance psychological resilience in the face of setbacks. This capacity is especially crucial for Chinese athletes who are subject to long-term high-intensity training and highly regulated environments.

### The moderating role of training years

The number of years athletes have engaged in training may moderate the relationship between SE and PASS, thereby influencing its underlying psychological mechanisms. In this sense, training years may serve as an important boundary condition in the association between SE and PASS. According to sociometer theory, SE functions as an internal monitor of one’s perceived social acceptance, and its role may be reinforced through long-term competitive experiences (Leary and Baumeister, 2000) Athletes with longer training years accumulate greater experience in team interactions and coach feedback (Kristiansen and Roberts, 2010), which may strengthen their ability to integrate external support cues based on SE. Furthermore, the broaden-and-build theory (Fredrickson and Branigan, 2005) suggests that positive emotions expand cognitive scope by building “psychological resources” over time. Similarly, long-term training may foster Decentering, enabling athletes to detach more effectively from negative thoughts (Bühlmayer et al., 2017). On this basis, the present study hypothesizes that training years positively moderate the effect of SE on PASS, such that the longer the training years, the stronger the positive predictive effect of SE on PASS.

**Figure 1:**
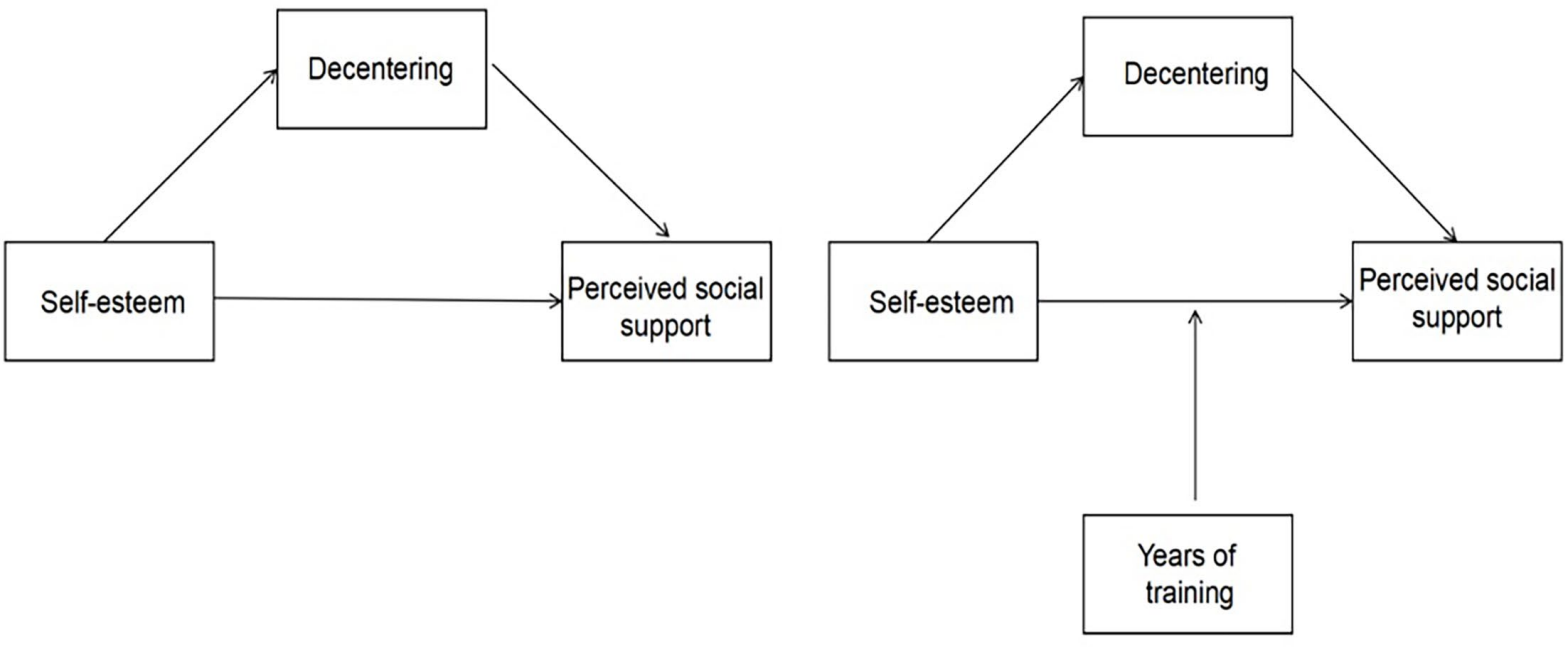
The research path.

In summary, sociometer theory explains the close relationship between SE and social support, while the Broaden-and-Build Theory provides a basis for understanding the role of decentering in emotional regulation and social adaptation. This allows for the derivation of a mediation model involving decentering. Existing research primarily focuses on the direct effects of social support, with insufficient attention paid to the underlying psychological mechanisms and pathways. Moreover, there is a lack of research specifically targeting athletes, particularly Chinese athletes, which provides a valuable entry point for this study. Based on this, the present study explores the moderating role of athletes’ training duration, offering a more specific view of the relationships between the variables. This study first examines the predictive effect of athlete SE on perceived social support, and the mediating effect of decentering, and then investigates whether athletes’ training duration moderates the relationship between SE and perceived social support. The hypothesized model is shown in Figure 1.

H1: SE is positively correlated with PASS and positively predicts PASS.

H2a: SE positively predicts Decentering.

H2b: Decentering positively predicts PASS.

H2: Decentering mediates the relationship between SE and PASS.

H3: Training years moderate the relationship between SE and PASS.

## Method

### Participants

The participants were specialized athletes from a sports university in Xi’an, totaling 377. After excluding 23 invalid questionnaires, 354 valid questionnaires remained. Among these, 108 were National First-Class athletes, and 246 were National Second-Class athletes. There were 210 male students and 144 female students. The average age was 20.39 ± 2.16 years. There were 40 individuals with 1–2 years of training, 57 with 3–4 years, 83 with 5–7 years, and 174 with more than 7 years. The sports disciplines included baseball, football, aerobics, basketball, volleyball, and track and field, as detailed in Table 1.

**Table 1:** Descriptive statistics.

Sports discipline	Frequency	Percent	C. percent
Baseball	22	6.2	6.2
Male	8	36.3	
Female	14	63.3	
American football	24	6.7	13.00
Male	13	54.2	
Women	11	45.8	
Aerobic dance	18	5	18.10
Male	8	44.4	
Women	10	55.5	
Basketball	61	17.2	35.39
Male	37	60.7	
Daughter	24	39.3	
Volleyball	56	15.8	51.2
Male	22	39.3	
Women	34	60.7	
Athletics	50	14.1	65.33
Male	38	76	
Women	12	24	
Martial arts	19	5.3	70.7
Male	8	42.1	
Women	11	57.9	
Badminton	36	10.1	80.86
Male	26	72.2	
Female	10	27.8	
Soccer	68	19.2	100
Male	50	73.5	
Women	18	26.5	
Total	354	100	
Male	210	59.3	
Women	144	40.6	

### Procedure

The study received ethical approval from Xi’an Physical Education University. Data was collected using questionnaires that the athletes completed. Participants were provided with information about the study and gave informed consent. Additionally, they were recruited by colleges and universities in our country through special programs, single recruitment of fresh high school graduates, or amateur sports school graduates. These individuals have participated in national, international, or professional-level competitions in their respective fields and achieved excellent results.

### Measure

#### Perceived available support

##### Perceived available support in sports questionnaire

The Perceived Available Support in Sports Questionnaire (PASS-Q) (Freeman et al., 2011) developed by Freeman et al. was used, with a total of 16 entries including four dimensions, namely emotional support, SE support, information support, and tangible support. A five-point Likert scale was used, with higher scores representing higher PASS. In this study, the overall Cronbach’s alpha coefficient for this questionnaire was 0.946, and the four sub-dimensions were 0.911 for emotional support, 0.931 for self-esteem support, 0.821 for information support, and 0.902 for tangible support.

#### Rosenberg self-esteem scale

##### Rosenberg RSS scale (Rosenberg Self-esteem scale)

Developed by Rosenberg (1965), there are 10 entries in total, with higher scores representing higher levels of self-esteem, which is now widely used with good reliability, and in the present study, the overall Cronbach alpha coefficient was 0.773.

#### The decentering scale for sport

The Decentering Scale for Sport (The Decentering Scale for Sport, DSS) (Zhang et al., 2016): There are a total of 12 items. Which uses a five-point Likert scale, with higher scores representing higher levels of decentering, has good reliability and validity. The Cronbach’s alpha coefficient for this questionnaire in this study was 0.915.

### Data analysis

This study employed SPSS 25.0 for descriptive statistics and correlation analysis of the data. The PROCESS model 4 in the SPSS extension program was utilized to test the mediation model involving athletes’ SE, Decentering, and PASS (Hayes, 2017). Subsequently, the moderating role of the athletes’ years of training in the relationship between SE and PASS was further examined using PROCESS model 5.

## Results

### Common method bias

The data for this study were collected by administering a questionnaire to the athletes, which raises the potential issue of common method bias. The common method bias test was conducted using Harman’s one-factor method, which identified seven factors with eigenvalues greater than 1. The first factor accounted for 30.87% of the variance, which is below the critical threshold of 40%.indicating that there is no serious common method bias in this study.

### Descriptive statistics and correlation analysis

Independent samples t-test showed that athletes of different genders showed significant differences in perceptual social support (t = 2.76, *p* < 0.001), SE (t = 2.22, *p* < 0.01), and Decentering (t = 2.00, *p* < 0.05), and that male students scored higher than female students. Athletes with different years of training showed a significant difference in perceptual social support (*F* = 2.86, *p* < 0.05) Athletes with high years of training scored more than athletes with low years of training. Athletes’ perceptions of social support and its four dimensions of emotional support, SE support, informational support, and tangible support were significantly and positively correlated with SE and Decentering, and the results of the descriptive statistics are shown in Table 2.

**Table 2:** Descriptive statistics of variables and their correlation coefficients (*n* = 354).

Variables	M	SD	1	2	3	4	5	6
1. PASS-Q	57.58	13.23						
2. RSES	14.06	4.28	0.24^**^					
3. DSS	38.95	8.99	0.36 ^***^	0.16^**^				
4. ES	14.85	3.91	0.86 ^***^	0.20 ^***^	0.33 ^***^			
5. SS	15.03	3.82	0.89 ^***^	0.36 ^***^	0.38 ^***^	0.76 ^***^		
6. IS	14.23	3.51	0.87 ^***^	0.19 ^***^	0.31 ^***^	0.65 ^***^	0.72 ^***^	
7. TS	13.48	4.26	0.81 ^***^	0.17 ^***^	0.22 ^***^	0.52 ^***^	0.56 ^***^	0.64 ^***^

PASS-Q, Perceived Available Support in Sports Questionnaire; RSES, Rosenberg Self-esteem Scale; DSS, The Decentering Scale for Sport; ES, Emotional support; SS, Self-esteem support; IS, Information support; TS, Tangible support. *p < 0.05, **p < 0.01, ***p < 0.001.

### Analysis of mediating effects

Athlete SE significantly and positively predicted Decentering (B = 0.3, 95% confidence interval of [0.08, 0.52, *p* < 0.01). Athlete Decentering significantly and positively predicted PASS (B = 0.48, 95% confidence interval [0.34, 0.62], *p* < 0.001). Athletes’ SE significantly and positively predicted PASS (B = 0.51, 95% confidence interval of [0.22, 0.81], *p* < 0.001) (shown in Table 3). The use of the PROCESS model 4, showed that Decentering of the self was a mediating variable in the athletes’ SE to improve PASS. The indirect path SE → DSS → PASS (β = 0.14, 95%CI [0.01, 0.31]) which did not include zero. This indicates that the path is significant (shown in Table 4). Consequently, athletes’ Decentering acts as a partial mediator in the relationship between SE and their perception of social support, accounting for 21.21% of the total effect.

**Table 3:** Mediation analysis of athletes’ DSS.

Variables	Predictor	*R*	*R* ^2^	*F*	df1	df2	β	B	SE	LLCI	ULCI	*t*
PASS-Q	Gender	0.34	0.12	9.07 ^***^	5.0	348.0	−0.126	−3.38^*^	1.46	−6.25	−0.52	−2.32
	Grade						−0.168	−1.93^***^	0.58	−3.07	−0.78	−3.31
	Class of athlete						−0.06	−1.72	1.57	−4.82	1.38	−1.09
	Years of training						0.099	1.25	0.66	0.06	−0.04	1.19
	RSES						0.213	0.66^***^	0.16	0.35	0.97	4.18
DSS	Gender	0.20	0.04	2.94^*^	5.0	348.0	−0.116	−2.13^*^	1.03	−4.16	−0.09	−2.06
	Grade						0.047	0.37	0.41	−0.44	1.18	0.89
	Class of athlete						−0.088	−1.71	1.11	0.13	−3.90	−1.54
	Yeas of training						−0.006	−0.52	0.46	−0.96	0.86	−0.11
	RSES						0.142	0.3^**^	0.11	0.08	0.52	2.68
PASS	Gender	0.47	0.22	16.21 ^***^	6.0	347.0	−0.088	−2.36	1.38	−5.07	0.36	−1.7
	Grade						−0.184	−2.1^***^	0.55	−3.18	−1.03	−3.83
	Class of athlete						−0.031	−0.89	1.49	−3.82	2.03	−0.6
	Years of training						0.101	1.27^*^	0.62	0.06	2.48	2.07
	RSES						0.166	0.51^***^	0.15	0.22	0.81	3.43
	DSS						0.329	0.48^***^	0.07	0.34	0.62	6.79

PASS-Q, Perceived Available Support in Sports Questionnaire; RSES, Rosenberg Self-esteem Scale; DSS, The Decentering Scale for Sport; ES, Emotional support; SS, Self-esteem support; IS, Information support; TS, Tangible support. *p < 0.05, **p < 0.01, ***p < 0.001.

**Table 4:** Analysis of mediating effects of DSS.

Effect	Coeff.	SE	LLCI	ULCI	Proportion (%)
Aggregate effect	0.66	0.16	0.35	0.97	
Direct effect	0.51	0.15	0.22	0.81	77.27
Indirect effect	0.14	0.08	0.01	0.31	21.21

### Moderating effects of years of training for athletes

**Figure 2:**
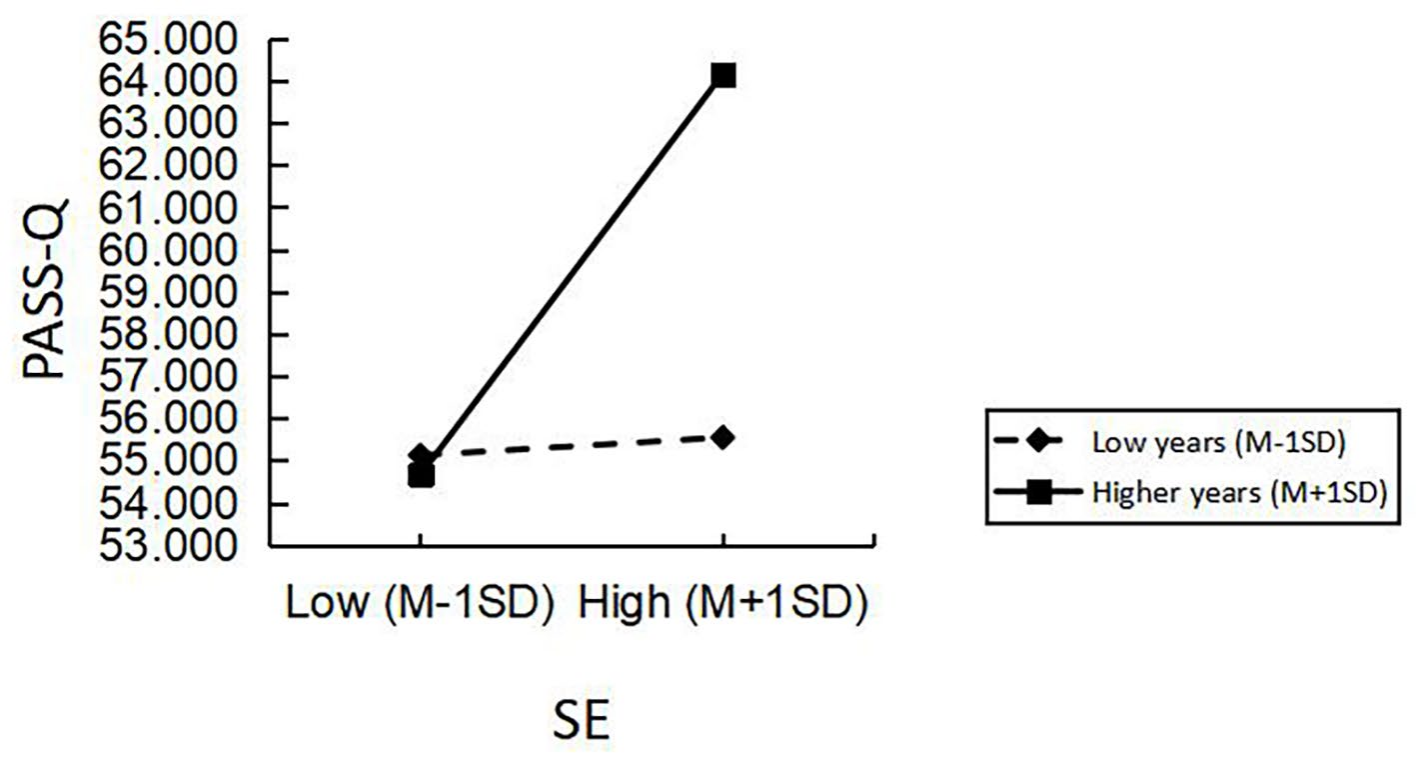
The moderating effect of training years on PASS.

Utilizing years of athlete training as the moderating variable within the mediation model, PROCESS model 5 was employed to examine the moderating effect while controlling for gender, age, grade, and sport level. The results, (shown in Table 5), indicate that the interaction between SE and years of athlete training was positively and significantly predictive of athletes’ comprehension of social support after incorporating years of athlete training into the mediation model (β = 0.55, β SE = 0.14, t = 3.98, *p* < 0.001). This suggests that years of athlete training exert a significant positive moderating effect on the mediating pathway of athletes’ SE affecting perceptual social support through Decentering (SE → Decentering →PASS). To visualize the moderating effects of the variables more intuitively, a further simple slope analysis was conducted (Figure 2).

**Table 5:** Examination of the moderating effect of athletes’ SE on PASS.

Model	Predictors	Coeff.	SE	*t*	LLCI ULCI
PSSQ	Gender	−2.03	1.36	−1.49	[−4.70, 0.64]
	Age	2.32	0.91	2.54^*^	[−9.43, −2.87]
	Grade	−6.15	1.67	−3.69 ^***^	[−9.43, −2.87]
	Class of athlete	−1.43	1.45	−0.98	[−4.28, 1.43]
	Years of training	2.10	0.65	3.21 ^**^	[0.82, 3.38]
	Self-esteem	0.55	0.14	3.98 ^***^	[0.28, 0.82]
	Years of training				
	*R^2^*	0.27			
	*F*	15.77			

PASS-Q, Perceived Available Support in Sports Questionnaire; RSES, Rosenberg Self-esteem Scale; DSS, The Decentering Scale for Sport; ES, Emotional support; SS, Self-esteem support; IS, Information support; TS, Tangible support. *p < 0.05, **p < 0.01, ***p < 0.001.

As can be seen from Figure 2, the moderating effect of low years of participation in training (M − 1SD) on the path of the relationship between athletes’ SE and perceptual social support is relatively small, while the moderating effect of high years of participation in training (M + 1SD) is relatively large, and the simple slopes for long years of training are more highly than those for low years of training. Comprehensive analyses can be inferred that the positive predictive effect of athletes’ SE level on PASS tended to increase gradually as athletes participated in the program for longer training years.

## Discussion

### Self-esteem and appreciation of the direct benefits of perceived available social support

The present study found that athletes of different genders, grades, and training years exhibited certain differences in self-esteem (SE), decentering (DSS), and perceived available social support (PASS). Male athletes scored significantly higher than female athletes in all three variables, a result consistent with previous findings. For example, Defreese and Smith reported that male athletes generally perceive higher levels of social support, which may stem from the fact that males in competitive sports are more likely to receive positive feedback from teammates and coaches (DeFreese and Smith, 2014) Differences in PASS between lower- and higher-grade athletes may be related to variations in their degree of team integration and competitive experience. Athletes with longer training years demonstrated higher PASS scores, aligning with the conclusion of Kristiansen and Roberts (Kristiansen and Roberts, 2010), who noted that long-term training participation facilitates the accumulation of interpersonal interaction experiences in competitive contexts, thereby enhancing sensitivity to and integration of support signals. No significant differences in SE, Decentering, or PASS were observed across different competition levels in this study. This contrasts with some prior research; for instance, Dahiya and Gupta argued that athletes at higher competitive levels typically exhibit stronger SE and resilience (Dahiya and Gupta, 2021). Such discrepancies may be attributable to sample characteristics: the participants in this study were primarily collegiate athletes rather than professional or international-level elite athletes, which may explain why differences by competition level did not emerge significantly. In sum, the demographic findings of this study are partly consistent with previous literature while also revealing new characteristics across different subgroups.

### The direct effect of self-esteem on perceived available social support

For athletes, self-esteem (SE) is a critical factor influencing athletic performance in competitive contexts. Athletes with higher levels of SE tend to demonstrate superior performance outcomes (Dahiya and Gupta, 2021). Previous research has also shown that individuals with higher SE possess a stronger ability to perceive support from various sources (Ikiz and Cakar, 2010), and the present study further corroborates this finding. Campbell et al. explained this phenomenon by noting that individuals with low SE are more likely to doubt their self-worth, engage in self-blame, experience distrusting relationships (Campbell et al., 2005), and exhibit heightened sensitivity to rejection (Downey et al., 2005). Drawing upon Sociometer Theory, which conceptualizes SE as a psychological gauge monitoring risks of social exclusion, such tendencies can be understood as follows: individuals with low SE, due to their hypersensitivity to “social rejection threats” may develop cognitive biases and defensive strategies in the perception of social support (Leary and Baumeister, 2000). Such defensive strategies may undermine the ability to perceive social support. The analysis confirms Hypothesis 2: SE is significantly and positively correlated with PASS, and SE serves as a significant positive predictor of athletes’ perceptions of available social support.

### The mediating effect of athletes’ decentering

Previous research has predominantly focused on the relationship between mindfulness and SE, or between Decentering and variables such as emotion regulation and mental health (Chandna et al., 2022; Rasmussen and Pidgeon, 2011), with relatively few studies directly examining whether SE can predict Decentering. The present study found that athletes’ SE positively predicted their Decentering levels. Athletes with higher SE were more likely to develop the capacity to “see thoughts as thoughts,” that is, to avoid equating transient cognitions or emotions with the self. This finding aligns with the broaden-and-build perspective, which posits that individuals with higher SE ruminate less and engage in fewer defensive cognitive processes, thereby approaching internal experiences with greater objectivity and openness—ultimately facilitating Decentering. Thus, Hypothesis H2a was supported. Research indicates that the higher an athlete’s level of decentering, the stronger their perception of social support, which aligns with the conclusion that “mindfulness promotes the sense of social support.” It also suggests that the ability to “maintain distance from self-experience” may help athletes overcome negative emotions. In competitive contexts, athletes can distance themselves from negative thoughts through decentering, making it easier to receive external support. Therefore, research hypothesis H2b is also supported. In summary, SE, as an individual resource, not only directly helps athletes expand their attentional breadth, allowing them to capture more cues for support, but also enhances their ability to detach from negative emotions through decentering, thereby reducing cognitive biases caused by excessive rumination or self-criticism and improving their capacity to perceive social support (Sun et al., 2019; Zhang et al., 2021), Collectively, these findings further confirmed Hypothesis H2.

It is also important to note that excessive, uncontrolled exercise may lead athletes to develop psychological dependence on the activity itself, manifesting as over-engagement or even “exercise addiction.” Exercise addiction reflects an individual’s excessive reliance on physical activity and an avoidance of negative emotions, potentially hindering the effective perception of social support. Decentering can assist individuals in detaching from negative experiences. Among college students, depression is significantly positively correlated with emotional exhaustion and smartphone addiction, with emotional exhaustion partially mediating the effect of depression on smartphone addiction. This finding suggests that negative emotions and the depletion of emotional resources are often key psychological mechanisms in the development of addiction patterns (Feng and Dou, 2024). If we apply this finding to the sports context, we can infer that when athletes lack effective emotional regulation strategies, depression and emotional exhaustion may exacerbate their over-dependence on training or competition, potentially evolving into exercise addiction. In contrast, decentering, as a positive cognitive processing technique, can help athletes detach from negative emotions, thereby interrupting this vicious cycle.

From another perspective, the construct of “Decentering” examined in this study also shares functional similarities with the concept of “flow experience” described in the literature. Both emphasize athletes’ ability to disengage from negative emotions or distracting cognitions and instead focus attention on the immediate experience, thereby fostering more positive psychological outcomes. The distinction lies in their emphases: Decentering highlights a cognitive shift toward adopting an objective, observer-like stance toward adverse events, whereas flow experience emphasizes immersion and enjoyment at the affective and motivational level. The two processes may contribute to athletes’ adaptive functioning through different pathways, suggesting that future research could integrate “Decentering” and “flow experience” within a unified model to illuminate the multidimensional mechanisms by which athletes sustain psychological health and social support perceptions in high-pressure environments (Hu et al., 2025).

### Realizing decentering based on Chinese and Western perspectives

The mechanism whereby SE influences PASS through Decentering in this study can be interpreted through the lens of cross-cultural integration. Sociometer theory highlights SE as an indicator of social acceptance (Leary and Baumeister, 2000), and its applicability has been widely validated in Western individualistic contexts. However, in Eastern collectivist cultures, SE is often shaped by external evaluations and collective honor, leading athletes to ground their sense of self-worth more heavily in others’ perceptions. Within this cultural context, the role of Decentering becomes particularly crucial: it enables athletes to disengage from the cognitive pattern of “performance = self-worth,” adopt a more objective perspective toward failures and setbacks, and thereby buffer the negative effects of external evaluative pressure. From the perspective of the broaden-and-build theory, Decentering not only facilitates the generation of positive emotions but may also function as a relational regulator in Eastern cultures. Chinese cultural traditions emphasize concepts such as *wu wo* (selflessness), harmony, and relational orientation, which align closely with the Decentering characteristic of viewing experiences from an observer perspective. Thus, in collectivist contexts, Decentering may operate not merely as an individual-level psychological mechanism but also as a culturally embedded adaptive strategy that helps athletes maintain harmonious social relationships and strengthen their perception of support. In Western mindfulness traditions, Decentering is often conceptualized in terms of “detached observation,” which alleviates psychological distress by treating thoughts as transient mental events (Bishop et al., 2004). By contrast, classical Confucian notions of *ke ji* (self-restraint) and Daoist principles of *ziran* (naturalness) frame Decentering as an ethical relational skill that promotes collective harmony (Hwang, 2012). Despite these divergent cultural emphases, the relationship between SE and Decentering reveals a deeper consistency in psychological adaptation. Whether in Western individualist contexts, where the primary aim is individual authenticity, or in Eastern collectivist settings, where the goal is relational harmony, the core function of these processes is to ensure that individuals feel worthy of social acceptance and maintain group connectedness (Leary and Baumeister, 2000). Moderate decentering-related behaviors—such as empathy and cooperation—enhance social acceptance within the group, which in turn reinforces SE. Higher SE further reduces defensive self-focus and enhances PASS, creating a positive feedback loop. Both cultural systems ultimately require maintaining this dynamic balance in order to sustain social adaptation.

### Moderating effect of years of training of athlete

This study found that training years positively moderated the predictive effect of SE on PASS (β = 0.55, *p* < 0.001), indicating that with increased training experience, athletes with higher SE demonstrated significantly greater PASS. Athletes who have participated in competitive sports for longer periods are more frequently exposed to team cooperation, coaching support, and social interactions within competition contexts (Kristiansen and Roberts, 2010), which may enhance their ability to recognize and integrate supportive signals from multiple sources. For example, athletes with longer training experience may be more likely to interpret a coach’s technical and tactical guidance as “professional support” rather than mere “criticism,” thereby strengthening their capacity to perceive support.

### Practical implications

In performance-oriented athletic training, coaches should not only focus on enhancing athletes’ competitive achievements but also place equal emphasis on their psychological well-being. Strengthening the development of athletes’ SE can, in turn, enhance their capacity to perceive social support (PASS), thereby facilitating improved athletic performance and better competitive outcomes. Furthermore, coaches may implement differentiated interventions tailored to athletes with varying training years. For athletes with shorter training experience, it is advisable to provide direct and explicit forms of support—such as emotional encouragement and tactical guidance—to help them establish a stable sense of SE. Conversely, for athletes with longer training experience, mindfulness-based approaches aimed at cultivating Decentering may be more effective, enabling them to reinterpret negative feedback as constructive tactical or technical guidance, thereby improving the internalization of perceived support. In addition, athletes who maintain closer relationships with their coaches are more likely to adopt positive attitudes and self-perceptions. Therefore, coaches should foster strong, supportive relationships with their athletes, enhancing their sense of attention and respect, which, in turn, strengthens PASS and motivates athletes to engage more positively in training and competition.

### Limitations

This study has several limitations. First, a cross-sectional design was used, which cannot reveal the causal relationships between the variables. Future research should adopt longitudinal tracking or experimental designs to verify the causal pathways among SE, decentering, and perceived social support. Second, the data primarily relied on self-report questionnaires, which may be influenced by social desirability bias or response bias. Future studies could incorporate multi-source data collection, such as coach and peer assessments, as well as behavioral observations, to enhance the reliability and validity of the conclusions. Third, the sample was limited to average-level Chinese athletes, lacking elite athletes. Future research could expand the sample to include athletes of different competitive levels to increase the external validity of the results. Finally, despite the use of methodological control and statistical tests, there is still the potential for common method bias. Future research could introduce additional data sources, such as multi-method data collection or experimental designs, to improve the reliability of the findings.

## Conclusion

This study examined the mechanisms through which SE promotes athletes’ PASS. The findings indicated that athletes’ SE not only positively predicted Decentering and PASS, but also positively predicted PASS through the mediating role of Decentering. Moreover, athletes’ training years significantly and positively moderated the pathway from SE to PASS. These results provide empirical support for understanding how SE enhances athletes’ PASS, as well as the underlying role of Decentering in this process.

## Data Availability

The original contributions presented in the study are included in the article/supplementary material, further inquiries can be directed to the corresponding author.
